# LINC00240 Knockdown Suppresses the Proliferation, Migration, and Invasion of Ovarian Cancer Cells Through the miR‐30c‐5p/P4HA2 Axis

**DOI:** 10.1111/jcmm.71171

**Published:** 2026-05-10

**Authors:** Yunjie Tian, Jianlei Wu, HaiBo Zhang

**Affiliations:** ^1^ Department of Gynecology The Fourth Hospital of Hebei Medical University Shijiazhuang China; ^2^ Department of Gynecological Oncology Shandong Cancer Hospital and Institute, Shandong First Medical University and Shandong Academy of Medical Sciences Jinan China

**Keywords:** LINC00240, miR‐30c‐5p, ovarian cancer, P4HA2, proliferation

## Abstract

Ovarian cancer is a particularly lethal malignancy often diagnosed at advanced stages, highlighting the urgent need for novel therapeutic strategies. This study investigates the expression and functional role of LINC00240, miR‐30c‐5p, and P4HA2 in ovarian cancer pathogenesis. Using datasets GSE66957 and the GEPIA database, we assessed LINC00240 expression levels and employed quantitative real‐time polymerase chain reaction (qRT‐PCR) to evaluate the expression of LINC00240, miR‐30c‐5p, and P4HA2 in ovarian cancer samples. Bioinformatics analysis via TargetScan software predicted interactions between these molecules, which were validated through dual‐luciferase reporter assays. Functional assays, including colony formation and Transwell assays, assessed the impact of LINC00240 and miR‐30c‐5p on cell proliferation, migration, and invasion. Our results indicate that LINC00240 and P4HA2 are significantly overexpressed, while miR‐30c‐5p is downregulated in ovarian cancer. Furthermore, LINC00240 modulates ovarian cancer malignancy by regulating P4HA2 expression through binding with miR‐30c‐5p. These findings elucidate the role of the LINC00240/miR‐30c‐5p/P4HA2 axis in ovarian cancer and suggest new avenues for targeted therapeutic interventions.

## 1 Introduction

Ovarian cancer is commonly diagnosed at a late stage and accounts for the highest number of deaths among all gynaecological cancers [1]. It shows diversity in molecular, cellular, and bodily appearance over time and space [2]. The disease is comprised of multiple subtypes, each defined by specific molecular features that evolve as the tumour progresses and acquires therapeutic resistance. This makes treating ovarian cancer challenging [3]. Unravelling the mechanisms of ovarian cancer will significantly support early diagnosis strategies and improve targeted therapies personalised for each individual, leading to better patient prognosis and survival.

Long non‐coding RNAs (lincRNAs) are a novel class of non‐coding RNA molecules (> 200 bases) that have essential role in various biological processes, including transcriptional regulation and chromatin remodelling as cell fate determinants affecting cellular function and disease progression [4]. LncRNAs add a further level of complexity to regulatory processes by working both at the genomic and epigenetic scale. Their functions are transcriptional and post‐transcriptional: they mediate nucleic acids‐proteins interactions that control gene expression in the cytoplasm as well as nucleus [5]. Studies about regulatory interactions (e.g., miRNA–mRNA, miRNA–miRNA or lncRNA) have been previously demonstrated [6, 7]. Additionally, many lncRNAs have been shown to involve in the occurrence and development of different cancer types [8, 9, 10]. However, there has been limited elucidation of the specific dynamics by which non‐coding RNAs are regulated. The interaction among lncRNAs, miRNAs, and mRNAs in the biological processes of cancer requires more profound exploration.

LINC00240 has emerged as a novel marker for preeclampsia [11] and also plays critical roles in the lncRNA‐mRNA crosstalk networks essential for the tumorigenesis of oesophageal and cervical cancers [12, 13]. However, the potential effects of LINC00240 in ovarian cancer remain unexamined.

P4HA2 is a member of the 4‐prolyl hydroxylase (P4H) family, and the role of P4HA2 in tumorigenesis and development has been reported. P4HA2 was highly expressed in breast cancer tissues and was associated with poor prognosis in patients. Knockdown or the use of inhibitors to reduce the expression of P4HA2 can significantly inhibit the growth and metastasis of tumour cells [14, 15]. However, the function of P4HA2 in ovarian tumour tissues is still unclear and needs further investigation.

In the present study, we identified a significant upregulation of LINC00240 in ovarian cancer tissues based on data from the GSE66957 dataset. We investigated the interplay between LINC00240, miR‐30c‐5p, and P4HA2 in ovarian cancer and found that LINC00240 regulates P4HA2 expression through binding with miR‐30c‐5p. This regulatory mechanism ultimately influences the growth, migration, and invasion of ovarian cancer cells. Our analysis of clinical samples revealed that LINC00240 is significantly overexpressed in ovarian cancer tissues, exhibiting a negative correlation with miR‐30c‐5p levels and a positive correlation with P4HA2 expression. These findings elucidate the role of LINC00240 in ovarian cancer pathogenesis and suggest its potential as a therapeutic target.

## 2 Materials and Methods

### 2.1 Cell Culture and Tissue Samples

OVCAR3 and SKOV3 cells were purchased from Hycyte Co. Ltd (Jiangsu, China). Trypsin, culture medium, and fetal bovine serum were all purchased from Gibco (Gibco, California, USA). OVCAR3 cells were cultured in RPMI‐1640 medium with 10% fetal bovine serum, 1% streptomycin/penicillin together with a final concentration of insulin at the dose of 0.1 mg/mL (Invitrogen, California, USA). SKOV3 cells were cultured in McCoy's 5A medium supplemented with 10% fetal bovine serum and 1% penicillin/streptomycin. All cells were incubated at 37°C in a humidified atmosphere containing 5% CO_2_.

All clinical specimens were obtained from the Fourth Hospital of Hebei Medical University, where every participant supplied written informed consent. The research was granted approval by the Ethics Committee at the Fourth Hospital of Hebei Medical University (Number: 2023KS157). The study adhered to the principles outlined in the Declaration of Helsinki. The basic pathological information of the patient is shown in Table S1.

### 2.2 Plasmids Construction

The complete sequences of LINC00240 were inserted into the pcDNA3.1 vector to conduct plasmids for LINC00240 overexpression. The mutant LINC00240 (LINC00240‐MUT) plasmid was constructed through site‐specific mutagenesis based on the wild‐type LINC00240 (LINC00240‐WT) plasmid. The mutant P4HA2 (P4HA2‐MUT) plasmid was constructed through site‐specific mutagenesis based on the P4HA2‐WT plasmid.

### 2.3 Cell Transfection

Target shRNAs including control were obtained through Genepharma (Shanghai, China) for LINC00240 and P4HA2. The shRNA construct targeting sequences are shown in Table 1. Synthetic oligonucleotides, including miRNA mimics and a negative control for the mimics (miR‐NC), as well as an inhibitor of miR‐30c‐5p, were procured from RiboBio (Guangzhou, China). For transfection, cells were plated in 6‐well plates at a density of 4 × 10^5^ cells per well 24 h before transfection. After that, cells were transfected with siRNA (50 nM) using Lipofectamine 2000 reagent (Life Technologies, CA, USA), according to the manufacturer's instructions. After transfection for 48 h, the cells were harvested, and some of them continued to be used for subsequent analysis.

**TABLE 1:** shRNA targeting sequences.

LINC00240‐homo‐1	S: 5′‐CAATAATGTTCGAAGTTAT‐3′
	A: 5′‐ATAACTTCGAACATTATTG‐3′
LINC00240‐homo‐2	S: 5′‐GGTTTGTTATACAGCAATA‐3′
	A: 5′‐TATTGCTGTATAACAAACC‐3′
LINC00240‐homo‐3	S: 5′‐GATAGTTTGTTATGCAATA‐3′
	A: 5′‐TATTGCATAACAAACTATC‐3′
P4HA2‐homo‐1	S: 5′‐CCTGATTTATGCAGAGAAA‐3′
	A: 5′‐TTTCTCTGCATAAATCAGG‐3′
P4HA2‐homo‐2	S: 5′‐GGATGGAGCAGGTGCTAAA‐3′
	A: 5′‐TTTAGCACCTGCTCCATCC‐3′
P4HA2‐homo‐3	S: 5′‐GGATCAAGGAGATCGCAAA‐3′
	A: 5′‐TTTGCGATCTCCTTGATCC‐3′
P4HA2‐homo‐4	S: 5′‐GAAGGTGACTACCGAACAA‐3′
	A: 5′‐TTGTTCGGTAGTCACCTTC‐3′

Abbreviations: A, antisense; S, sense.

### 2.4 Gene Datasets and Identification of Differentially Expressed Genes (DEGs)

Dataset GSE66957, comprised of normal samples (*n* = 12) and ovarian cancer samples (*n* = 57), was downloaded from the Gene Expression Omnibus (GEO) database (http://www.ncbi.nlm.nih.gov/geo). This dataset contains the largest number of instances and has the data already normalised. We used the GEO2R platform in our study to complete the differential gene expression analysis between ovarian cancer and normal samples; a threshold of |fold change| > 2 with *p* < 0.05 was chosen as DEGs. The downloaded files were preprocessed in the R software package, and we now run these codes for staking volcano plots and drawing heat maps. Steps included calibration, standardisation, and log2 transformation of the data.

### 2.5 GEPIA2

The GEPIA database (http://gepia2.cancer‐pku.cn/) serves as an online tool for gene expression pattern analysis and offers RNA sequencing data visualisation across tissues from the Genotype‐Tissue Expression (GTEx) project and The Cancer Genome Atlas (TCGA). It supplies this information to the Boxplot module, which then evaluates LINC00240 expression in TCGA‐OV (*n* = 426) and normal tissue (*n* = 88), with statistical analysis readouts from Student's *t*‐test. The “General” module provides an extensive pan‐cancer analysis of LINC00240, drawing on data obtained from the TCGA database. A statistical significance threshold of *p* < 0.05 is applied.

### 2.6 UALCAN

Analysis of cancer transcriptome data is accessible using the UALCAN database (http://ualcan.path.uab.edu/index.html). It provides researchers with functional gene expression from The Cancer Genome Atlas (TCGA) with the intent to assist in studying cancer biology or identifying biomarkers for diagnosis and treatment.

### 2.7 Cytoplasmic and Nuclear RNA Isolation

Cells were cultured to 80% confluence, washed twice with ice‐cold PBS, and lysed on ice for 5 min in cytoplasmic lysis buffer (0.1% NP‐40) to selectively disrupt the plasma membrane while maintaining nuclear integrity. The lysate was centrifuged at 500× *g* for 5 min (4°C), yielding the cytoplasmic fraction (supernatant) and the nuclear pellet. The pellet was resuspended in nuclear lysis buffer (1% SDS), vortexed for 30 s, and centrifuged at 12,000× *g* for 10 min (4°C) to collect the nuclear fraction (supernatant).

### 2.8 Fluorescence *In Situ* Hybridisation (FISH)

The Cy3‐labelled LINC00240 and FITC‐labelled miR‐30c‐5p mRNA probes were commercially synthesised by Sangon Biotech (Shanghai, China). For FISH, cells were sequentially treated with a protease K/pepsin digestion buffer, heat‐denatured at 75°C for 5 min, and hybridised with probe cocktail at 42°C overnight in a humidified dark chamber. Following stringent washes, nuclei were counterstained with DAPI, and fluorescence images were acquired using a confocal laser scanning microscope.

### 2.9 Quantitative Real‐Time Polymerase Chain Reaction (qRT‐PCR)

First, extract RNA from the cells, then reverse transcribe it into cDNA according to the instructions, and finally perform qRT‐PCR amplification using SYBR dye. The primer sequences used are listed in Table 2. The actual expression levels of the key genes were calculated by the 2^−ΔΔCT^ method.

**TABLE 2:** qRT‐PCR primer sequences.

Types	Primer sequences
P4HA2	F: 5′‐GGCTAAACACAGACTGGCCT‐3′
	R: 5′‐TCTCATCAGGGCTTTGGCAG‐3′
GAPDH	F: 5′‐AGGTGAAGGTCGGAGTCAACG‐3′
	R: 5′‐AGGGGTCATTGATGGCAACA‐3′
LINC00240	F: 5′‐TAAATTGCGGAGGCCAGAAGT‐3′
	R: 5′‐CCTCTGCAAACCATCGCAGT‐3′
U6	F: 5′‐CTCGCTTCGGCAGCACA‐3′
	R: 5′‐AACGCTTCACGAATTTGCGT‐3′
Hsa‐miR‐30c‐5p stem‐loop	5′‐GTCGTATCCAGTGCAGGGTCCGAGGTATTCGCACTGGATACGACGCTGAGAG‐3′
U6 stem‐loop	5′‐AACGCTTCACGAATTTGCGT‐3′
miR‐30c‐5p	F: 5′‐GCGCTGTAAACATCCTACACT‐3′
	R: 5′‐GCGTGTAAACATCCCCGACT‐3′
miR‐U6	F: 5′‐CTCGCTTCGGCAGCACA‐3′
	R: 5′‐AACGCTTCACGAATTTGCGT‐3′

Abbreviations: F, forward primer; R, reverse primer.

### 2.10 Cell Colony Formation Experiment

Cells were plated on culture dishes and maintained in a CO_2_ incubator for 2–3 weeks. The colonies were visible clumps in the culture dish at which point incubation was stopped. Then the supernatant was discarded, and cells were washed three times with PBS. They were then fixed in a 4% paraformaldehyde solution and stained with the crystal violet solution (Sigma, MO, USA) for an extended period, capped at approximately 20 min. The cells were washed three times with PBS to remove non‐bound dye after the staining step. Finally, the dye was washed off gradually by tap water and pictures were taken after drying.

### 2.11 Cell Migration and Invasion Assays

Cells were transfected with sh‐LINC00240‐2 for 24 h. Next, 2 × 10^5^ cells were seeded into Transwell inserts (8 μm pore size), and complete medium containing 15% serum was added to the lower chamber. Invading cells on the lower surface were fixed in 4% paraformaldehyde after a 24‐h incubation. They were stained with 0.1% crystal violet at room temperature for 1 min; the stain was afterward analysed using a Leica microscope. For the invasion assays, Matrigel (BD Biosciences, CA, USA) was applied to line the upper chamber before seeding cells, followed by a similar protocol to that shown for migration assays as described above. The number of migrated or invaded cells was counted in five randomly selected fields per chamber for three independent samples.

### 2.12 RNA Immunoprecipitation Assay (RIP)

Wash cells with PBS and lysed in RIP lysis buffer. Next, the lysates were incubated with MS2 antibody for an additional 2 h at 4°C. Following this step, each sample was combined with 30 μL of Protein A/G PLUS‐Agarose (Santa Cruz, CA, USA) and incubated at subzero temperatures for 4 h. Subsequently, the pellets were washed with PBS, brought up in 1 mL of TRIzol reagent, and a qRT‐PCR was performed on RNA that had been precipitated from its aqueous phase with specific primers to detect for binding products.

### 2.13 Luciferase Reporter Assay

To facilitate the relationship between LINC00240 and miR‐30c‐5p, we created WT luciferase reporter constructs, referred to as LINC00240‐WT, as well as mutant reporter constructs known as LINC00240‐MUT. This process involved incorporating either the WT or the MUT miR‐30c‐5p binding site into the 3′ untranslated region (UTR) of the pGL3 vector (Promega, WI, USA). Then, co‐transfection of LINC00240‐WT or LINC00240‐MUT with miR‐30c‐5p mimics in cells was performed using Lipofectamine 2000. At 48 h after transfection, luciferase activity was measured through a luciferase reporter assay.

### 2.14 Immunohistochemistry

Tissue samples embedded in paraffin blocks were used for immunohistochemistry. The paraffin block included cancerous and adjacent non‐cancerous tissues. The tissue sections were dehydrated and dewaxed, followed by antigen retrieval in 0.01 mol/L citrate buffered saline. Endogenous peroxidase activity was blocked with 3% H_2_O_2_ for 30 min. After that, the tissues were further incubated in 5% normal goat serum at room temperature for 1 h. Samples were then incubated with the P4HA2 antibody overnight. The following day, samples were incubated with an HRP‐conjugated secondary antibody at room temperature for 30 min before being exposed to a DAB staining solution. Haematoxylin solution of Mayer was employed to counterstain the nuclei. Following dehydration and clearing, coverslips were placed on the slides for images obtained under a microscope at 20× magnification.

### 2.15 Western Blot Assay

RIPA lysis was used to prepare lysates from whole cells. The supernatant was quantified by BCA protein concentration assay kit (Beyotime, Shanghai, China). Specifically, 20 μL of diluted supernatant and 200 μL of BCA working reagent were added to a 96‐well plate, incubated at 37°C for 30 min, and the absorbance was measured at 562 nm to calculate the protein concentration, ensuring that the protein amount was the same in each sample. Proteins were then separated using SDS‐PAGE and transferred onto PVDF membranes. The membranes were blocked with 5% non‐fat dairy milk and incubated with primary antibodies against P4HA2 (diluted 1:1000, CL0351, Abcam, Cambridge, UK) and β‐actin (diluted 1:5000, AC026, ABclonal, Wuhan, China) at 4°C overnight. Incubating with the Goat Anti‐Rabbit IgG H&L (HRP) (diluted 1:10,000, 511203, ZENBIO, NC, USA) for 2 h at room temperature. The PVDF membranes were then scanned, and the bands were quantified using a ChemiDoc MP Imager system (Bio‐Rad, CA, USA). The data are expressed as the mean ± SD, derived from a minimum of three separate experiments.

### 2.16 Statistical Analysis

Data analysis was performed using SPSS version 20.0 (SPSS Inc., IL, USA). Descriptive statistics in table were expressed as mean ± standard deviation for quantitative variables. Comparative *t*‐test was used for unpaired samples between groups. One‐way analysis of variance was performed for multi‐group comparisons. Pearson correlation analysis was used to examine potential associations between variables. *p* < 0.05 was considered statistically significant.

## 3 Results

### 3.1 LINC00240 Exhibits Elevated Levels in Ovarian Cancer Tissues

**FIGURE 1:**
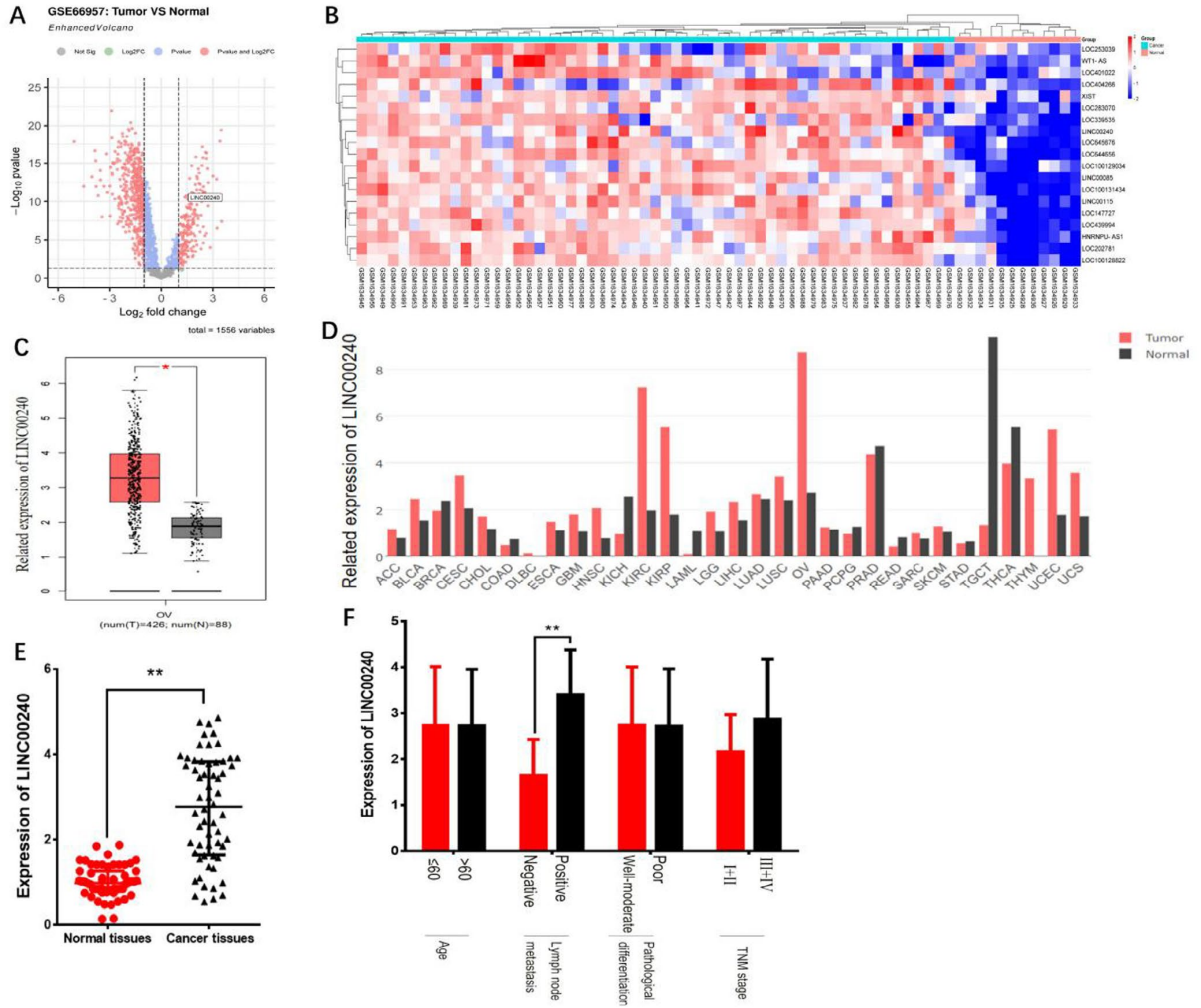
LINC00240 expression is enhanced in ovarian cancer tissues. (A, B) Volcano plots (A) and heatmap (B) of DEGs in ovarian cancer based on GSE66957 (57 samples of OV vs. 12 normal samples). (C) Analysis of the expression of LINC00240 in ovarian cancer based on 426 OV patients and 88 normal samples using the GEPIA2 database. (D) Pan‐cancer analysis of LINC00240 in various types of cancer. (E) LINC00240 levels in ovarian cancer tissues and adjacent cancer tissues (normal tissues). (F) Correlation between LINC00240 expression and various clinical characteristics. *p < 0.05; **p < 0.01.

To identify the potential candidate genes involved in ovarian cancer expression data, Affymetrix GeneChip dataset GSE66957 was downloaded from GEO database. Subseqnently, GEO2R was used to identify DEGs, followed by the generation of a volcano plot to visualise them (Figure 1A,B). Differential gene expression analysis of GSE66957 also revealed that LINC00240 was significantly upregulated in ovarian cancer (*n* = 57) as compared with normal ovary samples (*n* = 12) (Figure 1B). With the GEPIA2 database, we also found the expression of LINC00240 was higher in tumor tissues (*n* = 426) than in normal tissues (*n* = 88) (Figure 1C). Large scale pan‐cancer analyses indicated the higher expressed levels of LINC00240 across some cancers such as kidney renal clear cell carcinoma (KIRC), kidney renal papillary cell carcinoma (KIRP), ovarian serous cystadenocarcinoma (OV), thymoma (THYM) and uterine corpus endometrial carcinoma (UCEC). The strongest elevation of LINC00240 expression in ovarian cancer from those was shown in Figure 1D. Sixty ovarian cancer patients satisfying the surgical criteria were recruited from our hospital. RNAs were extracted from the cancer tissues and adjacent normal tissues. Next, qRT‐PCR analysis showed a significantly upregulated LINC00240 mRNA levels in cancer tissues, which was consistent with the analysis from TCGA and GEO databases (Figure 1E). We divided 60 ovarian cancer patients into different groups, including age, lymph node metastasis, TNM stage and pathological differentiation. There were no statistically significant differences found regarding age, pathological differentiation, or TNM staging. However, a notable correlation was established between the expression of LINC00240 and the presence of lymph node metastasis (Figure 1F). These findings indicate the possible correlation between LINC00240 upregulation and ovarian cancer progression, laying the foundation for further investigations in our subsequent experiments.

### 3.2 LINC00240 Knockdown Represses Cell Proliferation, Migration, and Invasion of Ovarian Cancer Cells

**FIGURE 2:**
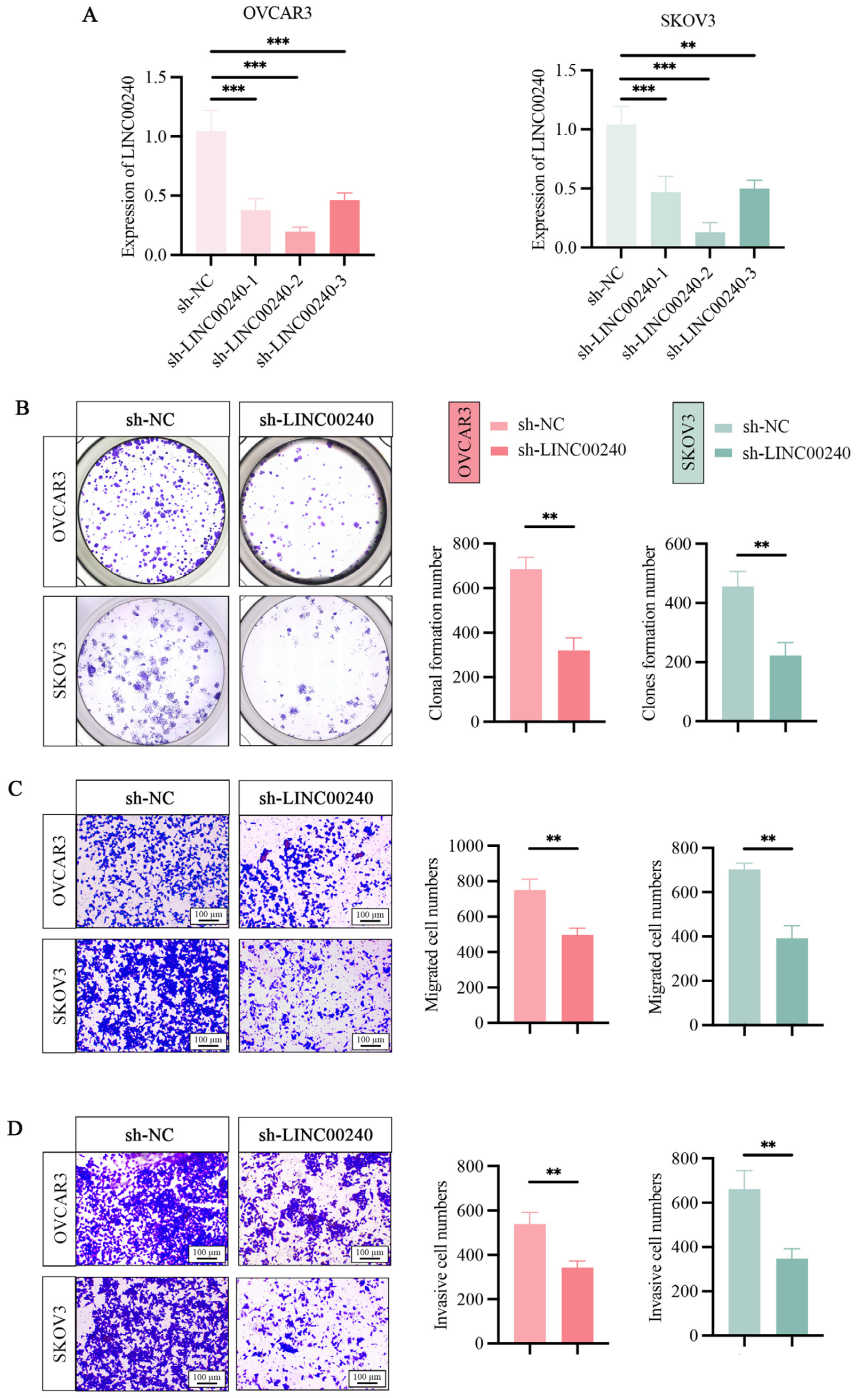
Knockdown of LINC00240 represses cell proliferation, migration, and invasion of OVCAR3 and SKOV3 cells. (A) Detection of knockdown efficiency of three different sh‐LINC00240 by qRT‐PCR. (B) Colony formation assay to detect clone formation ability of ovarian cancer cells after LINC00240 knockdown and subsequent analysis in number colonies formed. (C) Transwell migration assay after LINC00240 knockdown were performed in ovarian cancer cells, as well as statistical analysis of migrating cell numbers. (D) Transwell invasion assay demonstrated cell invasion ability of ovarian cancer cells under LINC00240 knockdown and statistical analysis of invasive cell numbers. **p < 0.01; ***p < 0.001.

The expression levels of LINC00240 were analysed by qRT‐PCR after transfecting with three different shRNAs in OVCAR3 and SKOV3 cells. All LINC00240 targeting shRNAs showed robust knockdown of LINC00240 in OVCAR3 and SKOV3 cells, with sh‐LINC00240‐2 exerting the most profound effect (Figure 2A). Therefore, sh‐LINC00240‐2 was selected for further experiments. Subsequently, we evaluated the effects of LINC00240 knockdown on the proliferation, migration, and invasion of OVCAR3 and SKOV3 cells. The cell colony formation assay showed that LINC00240 knockdown notably decreased colony formation of OVCAR3 and SKOV3 cells compared with the control group (Figure 2B). Moreover, LINC00240 knockdown also dramatically reduced the migration and invasion of OVCAR3 and SKOV3 cells compared with control cells, as observed in Transwell assays (Figure 2C,D).

### 3.3 LINC00240 Targets miR‐30c‐5p in Ovarian Cancer Cells

**FIGURE 3:**
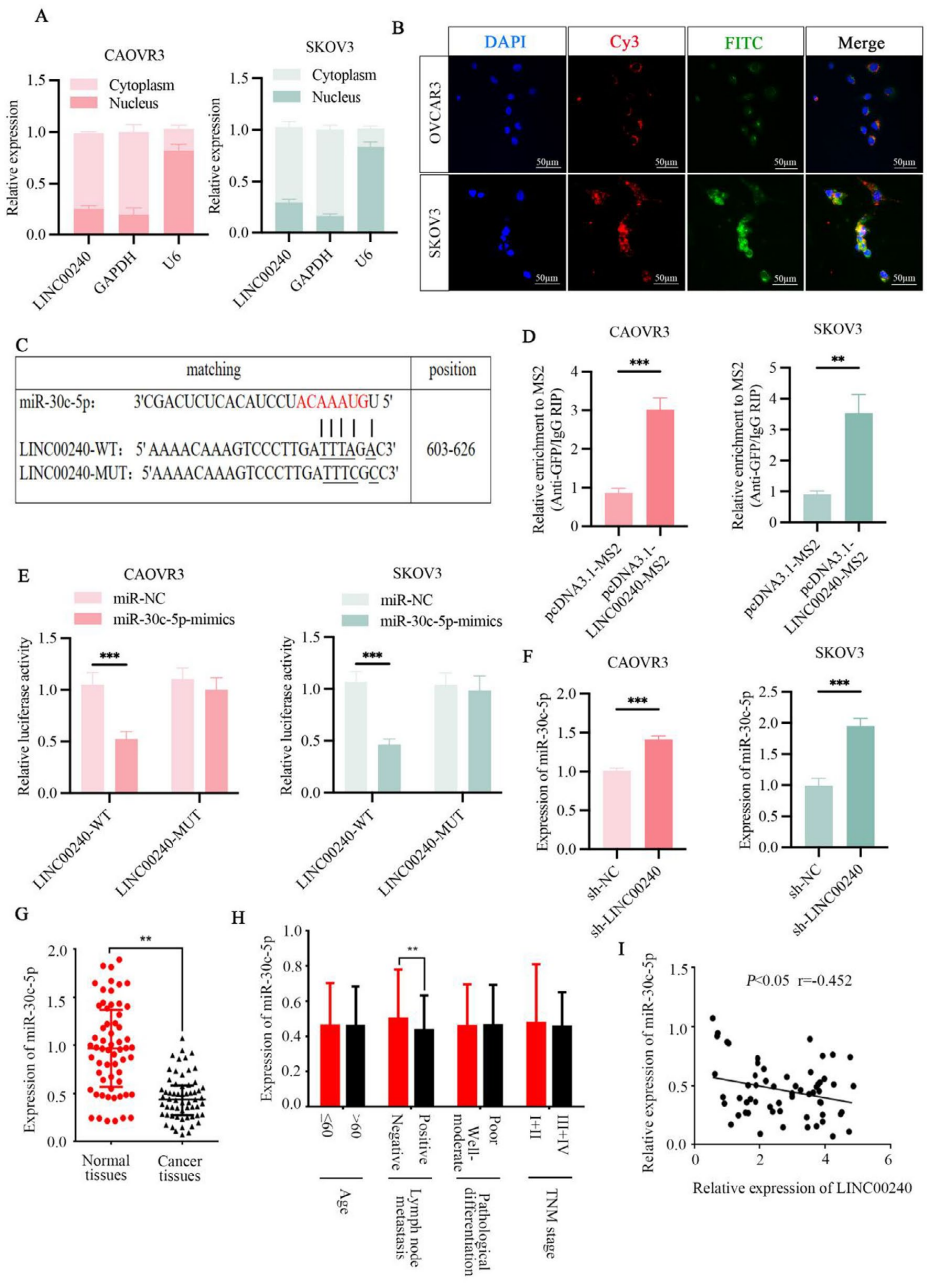
LINC00240 targets miR‐30c‐5p in ovarian cancer cells. (A) The mRNA expression levels of LINC00240 in the cytoplasm and nucleus of ovarian cancer cells. (B) Representative FISH images showing co‐localisation of LINC00240 (red) and miR‐30c‐5p (green) in ovarian cancer cells. Nuclei were counterstained with DAPI (blue). (C) miR‐30c‐5p and LINC00240 target prediction by TargetScan. (D) Ovarian cancer cells were transfected with pcDNA3.1‐LINC00240‐MS2 or the corresponding vector pcDNA3.1‐MS2. Next, an MS2‐RIP assay executed to detect miR‐30c‐5p levels. (E) The luciferase activity of WT or MUT LINC00240 reporter plasmids was measured in ovarian cancer cells after co‐transfection with either miR‐30c‐5p mimics or miR‐NC. (F) The mRNA expression levels of miR‐30c‐5p in ovarian cancer cells. (G) The mRNA expression levels transfection of miR‐30c‐5p in ovarian cancer tissues and adjacent cancer tissues (normal tissues). (H) Correlation between miR‐30c‐5p expression with various clinical characteristics. (I) The relevance of LINC00240 expression with miR‐30c‐5p. **p < 0.01; ***p < 0.001.

In order to assess the distribution of LINC00240 on OVCAR3 and SKOV3 cells, the expression of LINC00240 was examined in the cytoplasm and nucleus respectively, revealing a predominant localisation in the cytoplasm (Figure 3A). To further validate the interaction between LINC00240 and miR‐30c‐5p, we performed FISH using a Cy3‐labelled LINC00240‐specific probe and a FITC‐labelled miR‐30c‐5p probe in OVCAR3 and SKOV3 cells. The results demonstrated clear co‐localisation of these two molecules in the cytoplasm of OVCAR3 and SKOV3 cells (Figure 3B). Furthermore, TargetScan software predicts that LINC00240 contains binding sites for miR‐30c‐5p (Figure 3C). RIP experiments using an MS2 antibody demonstrated an interaction between LINC00240 and miR‐30c‐5p in OVCAR3 and SKOV3 cells (Figure 3D). To further confirm the direct interaction between LINC00240 and miR‐30c‐5p, we created both LINC00240‐WT and LINC00240‐MUT vectors. The findings from dual luciferase reporter assay revealed a significant reduction in relative luciferase activity in OVCAR3 and SKOV3 cells that were co‐transfected with LINC00240‐WT and miR‐30c‐5p mimics, compared to those that received the negative control vector. In contrast, there was no notable change in relative luciferase activity in OVCAR3 and SKOV3 cells that were co‐transfected with miR‐30c‐5p mimics and the LINC00240‐MUT vector (Figure 3E). Furthermore, in OVCAR3 and SKOV3 cells, siRNA‐mediated knockdown of LINC00240 significantly increased the mRNA expression of miR‐30c‐5p (Figure 3F). To further investigate the role of miR‐30c‐5p in ovarian cancer, its expression was assessed in ovarian cancer tissues and normal ovarian tissues, revealing a significant reduction in ovarian cancer samples (Figure 3G). Stratification of patients showed that miR‐30c‐5p levels were markedly lower in those with lymph node metastasis (Figure 3H). Notably, LINC00240 expression was inversely correlated with miR‐30c‐5p levels in ovarian cancer patients (Figure 3I). Collectively, these results indicate that LINC00240 can directly bind to miR‐30c‐5p and suppress its expression.

### 3.4 miR‐30c‐5p Expression in Ovarian Cancer Cells Is Negatively Linked With Cell Proliferation, Migration, and Invasion

**FIGURE 4:**
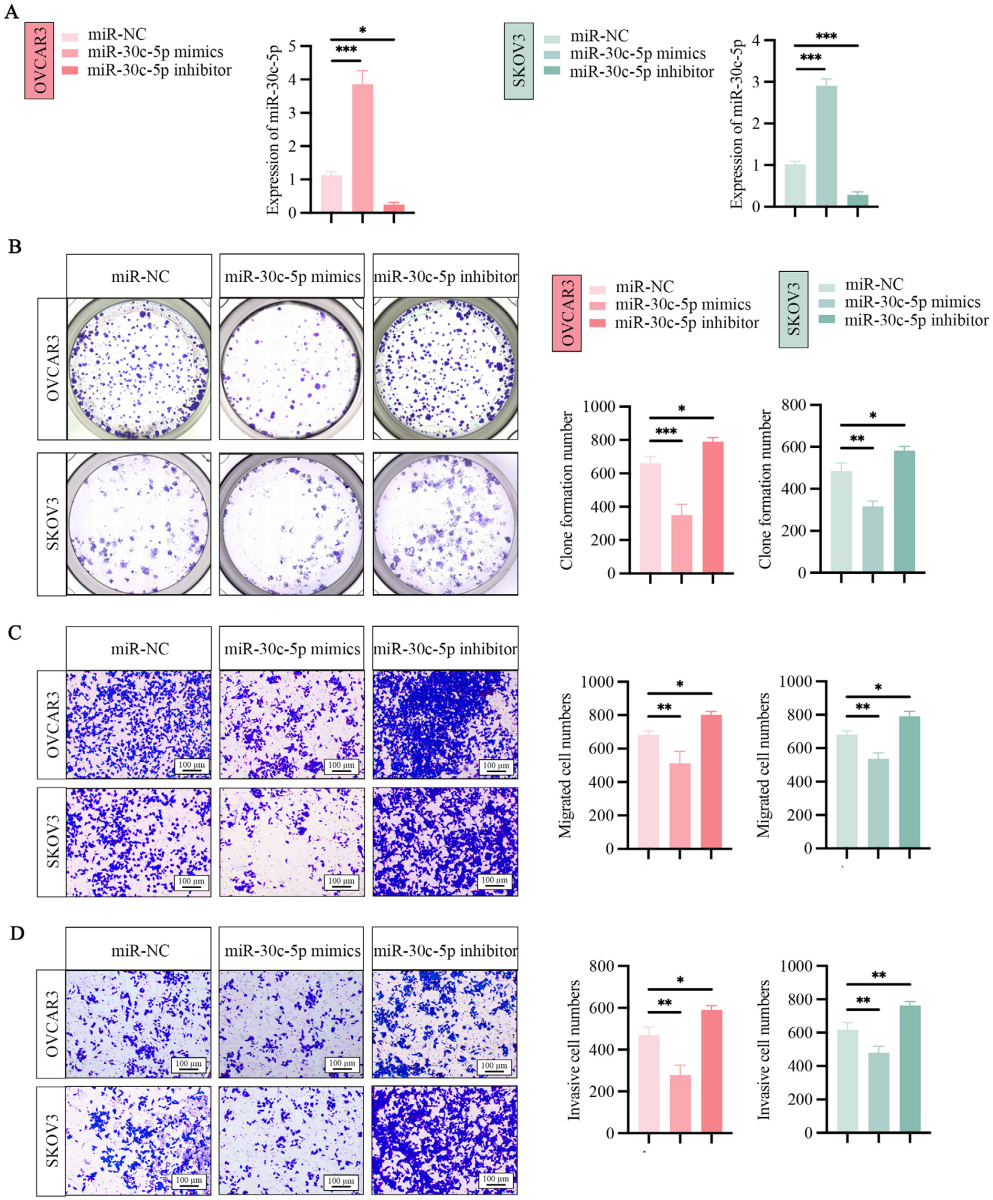
miR‐30c‐5p expression is negatively linked to ovarian cancer cell proliferation, migration, invasion. (A) qRT‐PCR was conducted to evaluate the transfection efficiency of miR‐30c‐5p mimics or inhibitor in OVCAR3 and SKOV3 cells. (B) The effects of miR‐30c‐5p mimic or inhibitor on the OVCAR3 and SKOV3 cell clone forming ability in OVCAR3 and SKOV3 cells were evaluated by colony formation assay and subsequent analysis in number colonies formed. (C) Transwell migration assays were performed after transfection with miR‐30c‐5p mimics or inhibitor, and migrating cell numbers were statistically analysed. (D) Transwell invasion assay demonstrated cell invasion ability under miR‐30c‐5p mimics or inhibitor, as well as statistical analysis of invasive cell numbers. *p < 0.05, **p < 0.01, ***p < 0.001.

qRT‐PCR was conducted to assess the expression levels of miR‐30c‐5p in ovarian cancer cells that were transfected with either miR‐30c‐5p mimics or a miR‐30c‐5p inhibitor. The results revealed a significant increase in miR‐30c‐5p expression after the introduction of the miR‐30c‐5p mimics. Conversely, the use of the miR‐30c‐5p inhibitor led to a marked decrease in the expression of miR‐30c‐5p within the OVCAR3 and SKOV3 cells (Figure 4A). After miR‐30c‐5p mimics or miR‐30c‐5p inhibitor transfection, OVCAR3 and SKOV3 cells were examined for their proliferation, migration, and invasion ability. The results from the colony formation assays indicated that overexpressing miR‐30c‐5p significantly reduced the ability of OVCAR3 and SKOV3 cells to form colonies compared to the control group. Conversely, lowering the expression of miR‐30c‐5p increased the colony formation capacity (Figure 4B). Additionally, Transwell assays shed light on how miR‐30c‐5p affects the migratory and invasive characteristics of the cells. The findings showed that introducing miR‐30c‐5p mimics into OVCAR3 and SKOV3 cells led to a marked decrease in both their migratory and invasive abilities compared to the control group. In contrast, using a miR‐30c‐5p inhibitor significantly boosted the migratory and invasive potential of OVCAR3 and SKOV3 cells compared to the control group (Figure 4C,D).

### 3.5 P4HA2 Is Elevated in Ovarian Cancer

**FIGURE 5:**
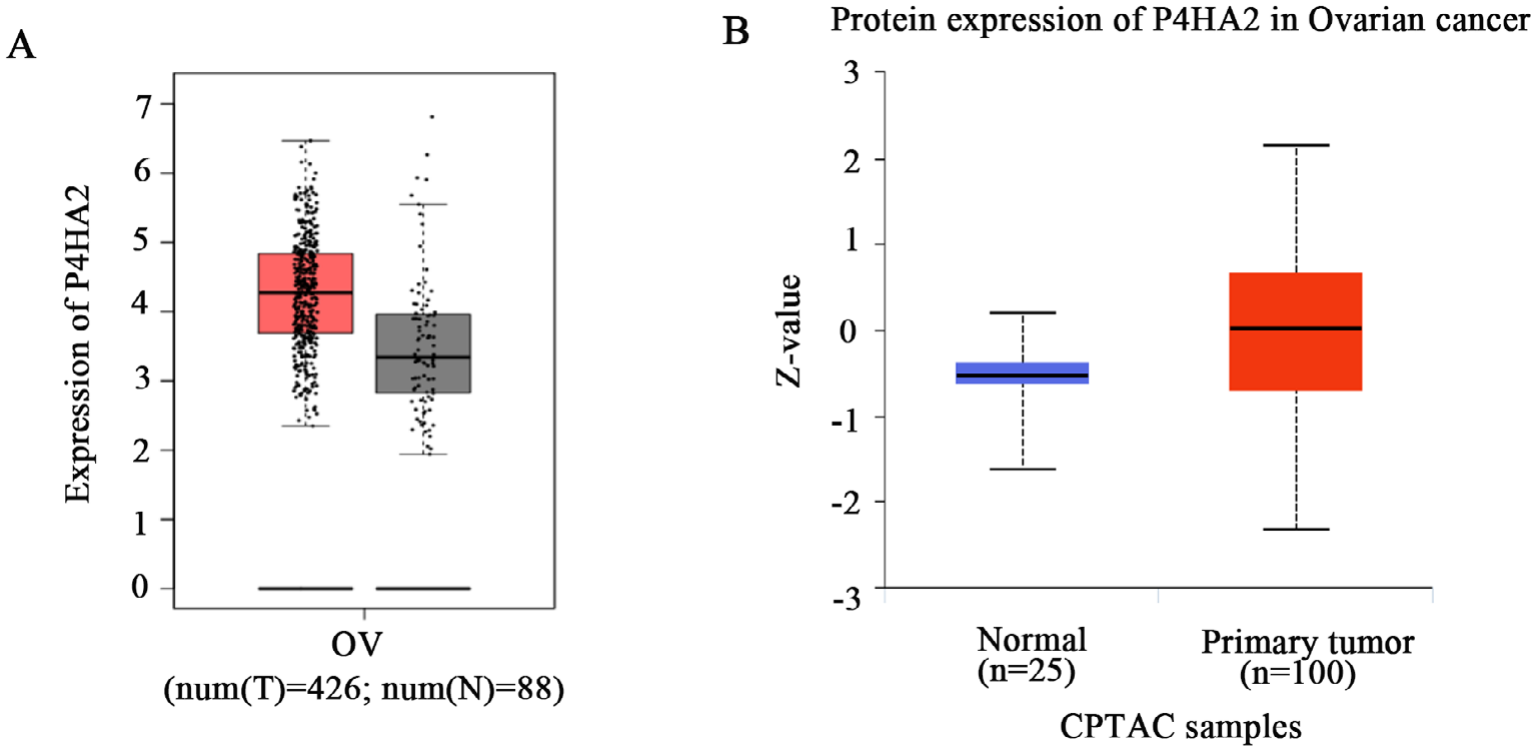
P4HA2 is elevated in ovarian cancer. (A) Analysis of P4HA2 expression in ovarian cancer obtained from GEPIA2, a database including 426 ovarian cancer patients and 88 normal samples. (B) The CPTAC database shows a high expression levels of P4HA2 in ovarian cancer tissues.

The relationship of P4HA2 expression with ovarian cancer progression was evaluated through GEPIA2. The results showed that P4HA2 expression was significantly increased in ovarian cancer tissues (Figure 5A). Consistently, an analysis of protein expression using data from the Clinical Proteomic Tumor Analysis Consortium (CPTAC) showed that the protein levels of the P4HA2 were significantly higher in ovarian cancer tissues compared to normal tissues (Figure 5B).

### 3.6 P4HA2 Is Identified as a Target Gene of miR‐30c‐5p

**FIGURE 6:**
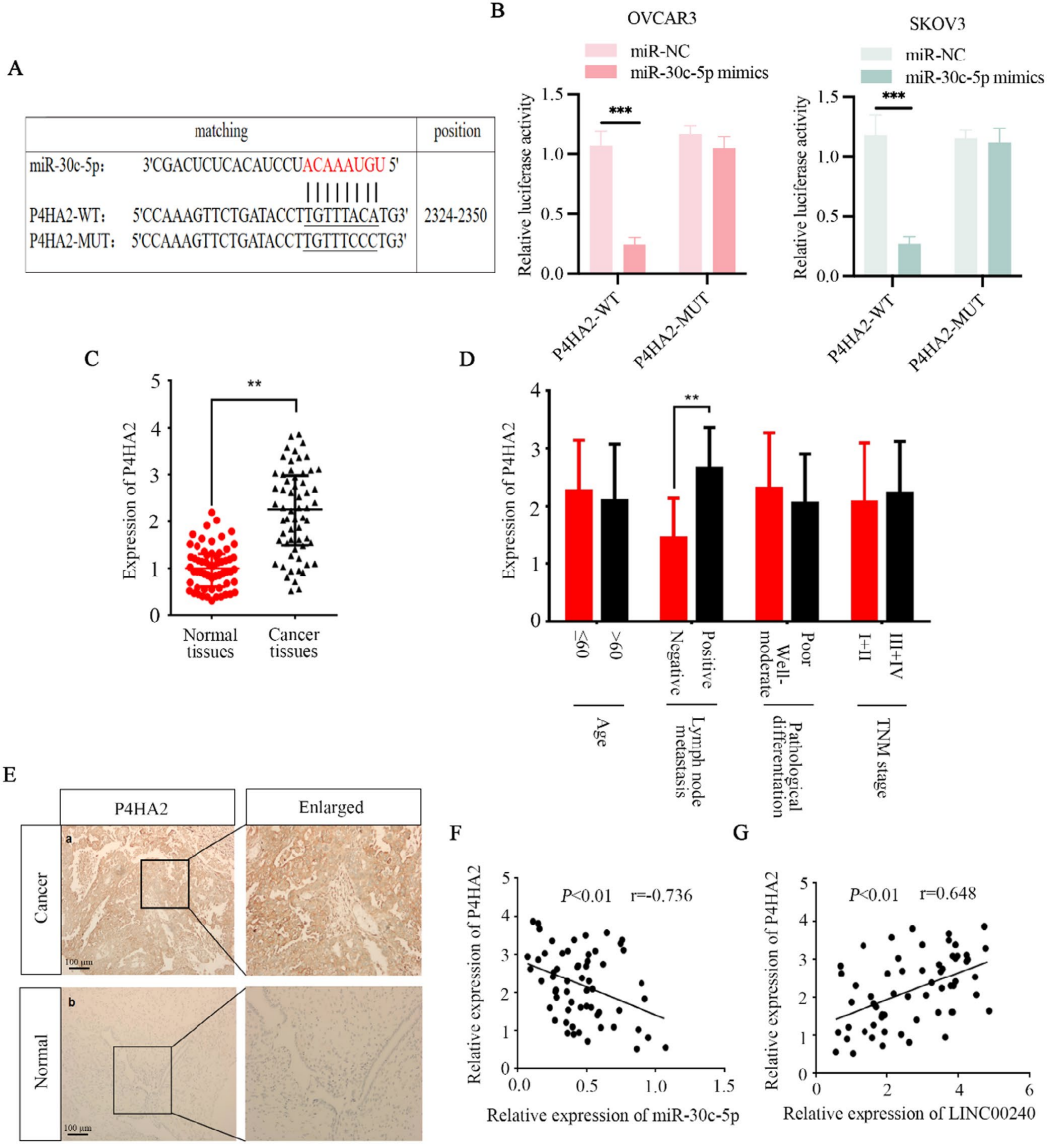
P4HA2 is a target gene of miR‐30c‐5p. (A) TargetScan was used to predicted binding regions of miR‐30c‐5p and P4HA2. (B) The luciferase activity of WT or MUT P4HA2 reporter plasmids was measured in ovarian cancer cells after co‐transfection with either miR‐30c‐5p mimics or miR‐NC. (C) P4HA2 levels in ovarian cancer tissues and normal ovarian tissues. (D) Correlation between P4HA2 expression with various clinical characteristics. (E) Immunohistochemical analysis of P4HA2 in cancer and adjacent cancer tissues (normal tissues). (F) The association between P4HA2 expression and miR‐30c‐5p expression. (G) The correlation between P4HA2 expression and LINC00240 expression. **p < 0.01.

Targetscan software indicates the bind site of miR‐30c‐5p and P4HA2 (Figure 6A). A pGL3 luciferase reporter containing the full‐length P4HA2 sequence (P4HA2‐WT) and a mutant P4HA2 sequence (P4HA2‐MUT) was constructed. Next, dual luciferase reporter assays confirmed the interaction between P4HA2 and miR‐30c‐5p. The results demonstrated that miR‐30c‐5p significantly suppressed the activity of LUC‐P4HA2‐WT in OVCAR3 and SKOV3 cells but did not change LUC‐P4HA2‐MUT activity (Figure 6B). Due to the above results, qRT‐PCR was also performed on clinical ovarian cancer specimens for validation. In clinical samples, P4HA2 was highly expressed in cancer tissues than in normal ovarian tissues (Figure 6C) and associated with lymph node metastasis (Figure 6D). Immunohistochemical analysis of P4HA2 in ovarian tissues demonstrated that cancerous tissues exhibited higher expression levels of P4HA2 compared to normal ovarian tissues (Figure 6E). Correlation analysis among P4HA2, miR‐30c‐5p, and LINC00240 in ovarian cancer samples revealed a negative correlation between P4HA2 expression and miR‐30c‐5p (Figure 6F), while we noted a positive association between P4HA2 and LINC00240 (Figure 6G).

### 3.7 P4HA2 Knockdown Significantly Attenuates Cell Proliferation, Migration, and Invasion of Ovarian Cancer Cells

**FIGURE 7:**
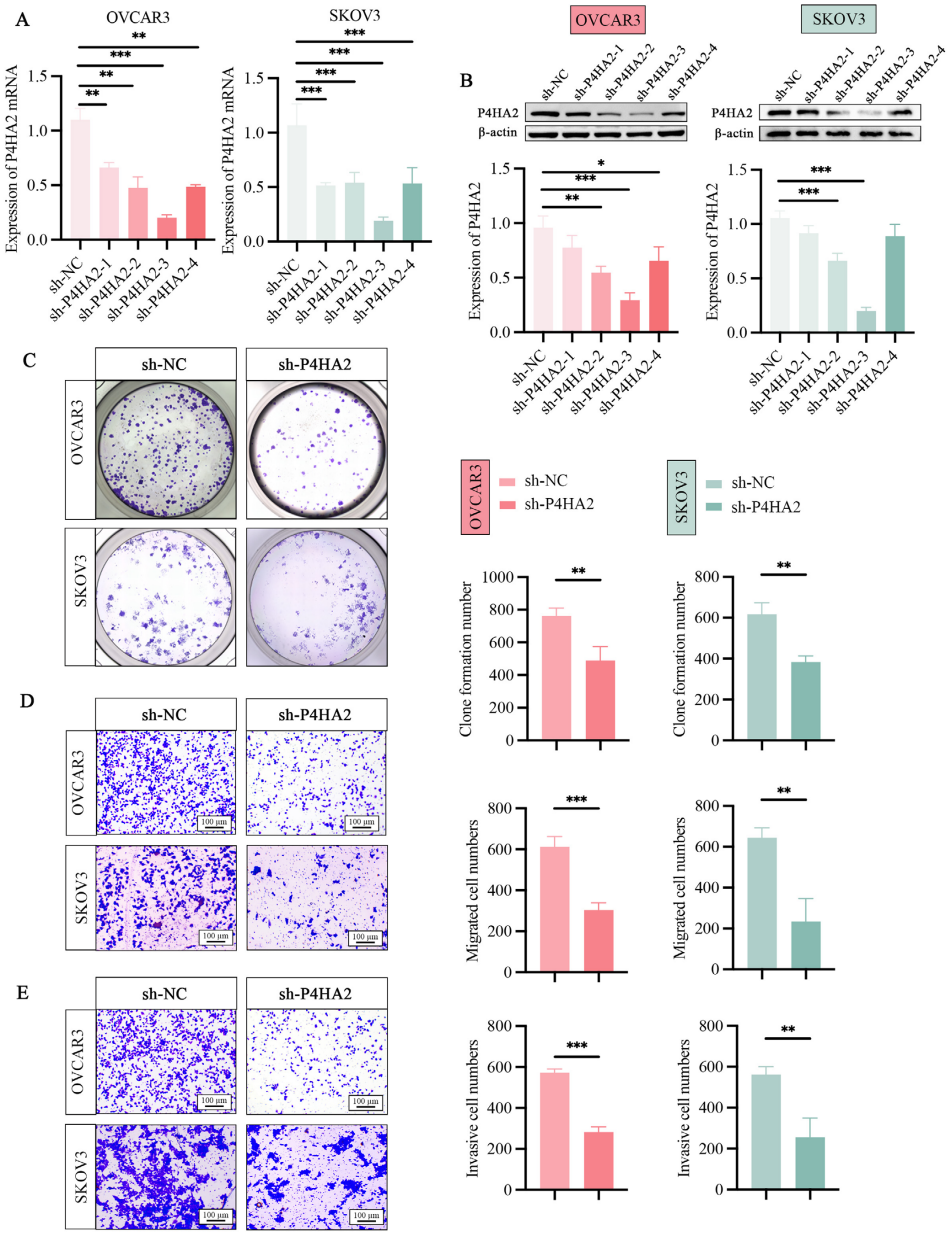
P4HA2 knockdown significantly attenuates cell proliferation, migration, invasion of ovarian cancer cells. (A) qRT‐PCR analysis to confirm knockdown efficiency of the three sh‐P4HA2s in ovarian cancer cells. (B) Western blot analysis to confirm knockdown efficiency of the three sh‐P4HA2s in ovarian cancer cells. (C) The impact of P4HA2 knockdown on the clone forming capacity of ovarian cancer cells was assessed through a colony formation assay and subsequent analysis of colony formation number. (D, E) The impact of P4HA2 knockdown on the cell migration (D) and invasion (E) ability of ovarian cancer cells was assessed through transwell assay and subsequent analysis of migrating and invasive cell numbers. *p < 0.05, **p < 0.01, ***p < 0.001.

Four distinct shRNAs targeting P4HA2 were designed for silencing P4HA2 expression in OVCAR3 and SKOV3 cells, with sh‐P4HA2‐3 demonstrating the most pronounced reduction in P4HA2 expression (Figure 7A). Western blot analysis demonstrated that shRNA‐mediated knockdown of P4HA2 significantly reduced P4HA2 protein expression levels in OVCAR3 and SKOV3 cells, which further validated the corresponding changes observed at the mRNA level (Figure 7B). Consequently, sh‐P4HA2‐3 was chosen for further investigation. The colony formation and Transwell assays have shown that the silencing of P4HA2 significantly impaired cell proliferation, migration, and invasion of OVCAR3 and SKOV3 cells (Figure 7C–E).

### 3.8 LINC00240/miR‐30c‐5p/P4HA2 Axis Promotes Cell Proliferation, Migration, and Invasion of Ovarian Cancer Cells

**FIGURE 8:**
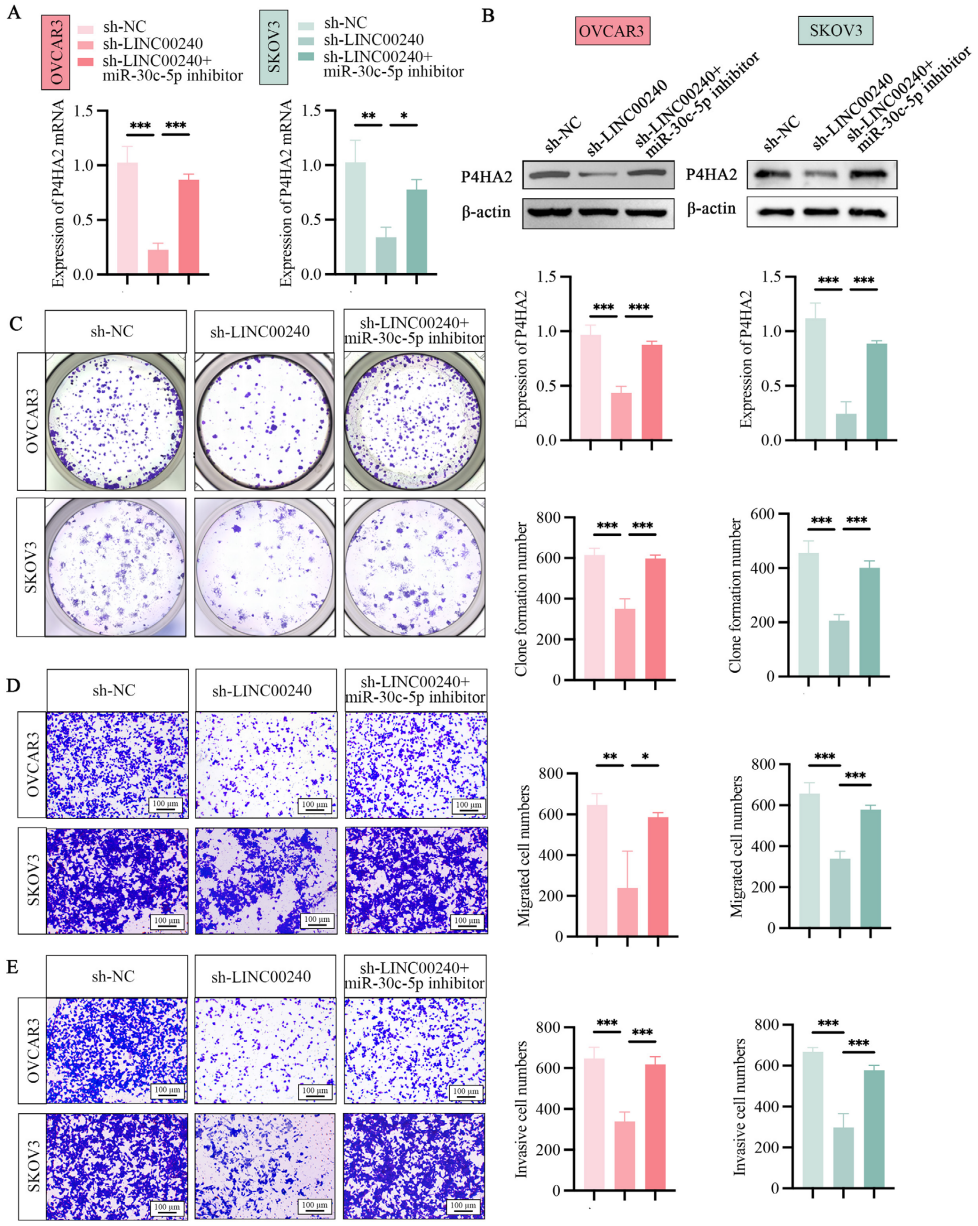
MiR‐30c‐5p and LINC00240 collaboratively regulate P4HA2 expression and contribute to ovarian cancer development. (A) The impact of co‐transfecting sh‐LINC00240 along with a miR‐30c‐5p inhibitor on the mRNA expression level of P4HA2 in ovarian cancer cells was assessed using qRT‐PCR. (B) The impact of co‐transfecting sh‐LINC00240 along with a miR‐30c‐5p inhibitor on the expression level of P4HA2 in ovarian cancer cells was assessed using Western blot. (C) The effects of simultaneous transfection with LINC00240 and miR‐30c‐5p on the clone formation ability of ovarian cancer cells was assessed through a colony formation assay, which involved analysing the number of clones that formed. (D) The migration of ovarian cancer cells was detected by Transwell migration assay under sh‐LINC00240 and miR‐30c‐5p inhibitor transfection, as well as statistical analysis of migrating cell numbers. (E) The invasion of ovarian cancer cells was assessed using Transwell invasion assay following transfecting with LINC00240 and miR‐30c‐5p, including statistical analysis of invasive cell numbers LINC00240. *p < 0.05, **p < 0.01, ***p < 0.001.

To further explore the interaction between LINC00240, miR‐30c‐5p and P4HA2, OVCAR3 and SKOV3 cells were transfected with sh‐LINC00240 in combination with miR‐30c‐5p mimics. We noted a substantial decrease in P4HA2 expression following transfection with sh‐LINC00240. However, when sh‐LINC00240 and miR‐30c‐5p inhibitor were co‐transfected into OVCAR3 and SKOV3 cells, the expression levels of P4HA2 had been restored (Figure 8A). Western blot analysis further confirmed the alterations at the protein level that corresponded to the mRNA expression changes (Figure 8B). Functional assays indicated that reducing LINC00240 levels resulted in decreased cell proliferation and migration abilities in OVCAR3 and SKOV3 cells (Figure 8C–E). Moreover, the miR‐30c‐5p inhibitor blocked the reduction in proliferation, migration and invasion of OVCAR3 and SKOV3 cells caused by LINC00240 knockdown (Figure 8C–E). These results suggest that LINC00240 knockdown‐induced inhibition of proliferation, migration and invasion in OVCAR3 and SKOV3 cells is partly mediated via the miR‐30c‐5p/P4HA2 signalling axis.

## 4 Discussion

Numerous studies have identified LINC00240 as a contributor to the progression of different types of cancer, operating through a miRNA‐dependent mechanism. For instance, LINC01232 facilitates gastric cancer progression by participating in the miR‐506‐5p/PAK1 axis [16]. Additionally, LINC00240 regulates nasopharyngeal carcinoma cell invasion through the miR‐26a‐5p/EZH2 axis [17]. In this study, we identified a novel miRNA that interacts with LINC00240, namely miR‐30c‐5p, demonstrating that LINC00240 could mediate ovarian cancer progression by regulating miR‐30c‐5p. We further investigated the precise mechanisms by which LINC00240 affects ovarian cancer.

Research has suggested that miR‐30c may function as a tumour suppressor [18]. The relationship between miR‐30c‐5p and BCL9 is essential in ovarian cancer as well as multiple myeloma [19, 20]. Additionally, inhibitors of miR‐30a, 30c, and 30d were able to prevent EMT induced by TGF‐β in ovarian cancer [21, 22]. Furthermore, miR‐30c‐5p is previously reported to involve in the TGF‐β and PI3K‐Akt signalling pathways [23]. This study discovered that LINC00240 can target miR‐30c‐5p, thereby regulating its expression. Beyond lncRNAs, hsa_circ_0000384 has been shown to mediate the level of TFDP1 through sequestering miR‐30c‐5p [24]. Therefore, further in vitro and in vivo evidence is needed to understand the precise regulatory pathways of miR‐30c‐5p in ovarian cancer.

MiRNAs regulate target mRNAs by suppressing their expression. Importantly, it has been proven that miR‐30c‐5p influences multiple gene targets. The recent study has suggested that PIK3CD could bind to miR‐30c‐5p directly and inhibit the expression of this miRNA in lung cancer [25]. Additionally, NORAD can reverse the injury of lung endothelial cells under LPS stimulation by the miR‐30c‐5p‐LDHA‐aerobic glycolysis pathway [26]. In this study, we found a new target gene P4HA2 of miR‐30c‐5p in ovarian cancer. The P4HA2 expression regulated the proliferation, migration, and invasion of ovarian cancer cells. We further explored their interactions and regulatory effects on each other with LINC00240, miR‐30c‐5p, and P4HA2, proposing that the action of LINC00240 might lie in a miR‐30c‐5 p/P4HA2 axis.

This study has several limitations. One limitation is the absence of in vivo animal models to validate the role of LINC00240 in tumorigenesis and its regulatory relationship with miR‐30c‐5p and P4HA2. Nevertheless, to support our in vitro findings, we analysed clinical ovarian cancer tissue samples, which yielded consistent results. Specifically, LINC00240 and P4HA2 expression levels were significantly elevated in ovarian cancer tissues compared with adjacent non‐tumour tissues, whereas miR‐30c‐5p expression was markedly reduced. Correlation analyses further demonstrated a positive association between LINC00240 and P4HA2 expression and a negative association between LINC00240 and miR‐30c‐5p levels in clinical samples. Another limitation concerns the gene knockdown experiments targeting LINC00240. Although sh‐LINC00240‐2 produced robust effects, the phenotypic consistency of the other two shRNAs (sh‐LINC00240‐1 and sh‐LINC00240‐3) was not assessed in this study. To enhance experimental rigour and strengthen the reliability of our conclusions, future work will include phenotypic validation using these additional shRNAs, thereby minimizing potential off‐target effects and providing a more comprehensive evaluation of LINC00240 function. Collectively, these findings support our in vitro conclusions and warrant further investigation.

Notably, we found that the expression levels of LINC00240, miR‐30c‐5p, and P4HA2 showed significant correlations with lymph node metastasis. LINC00240 functions as a competitive endogenous RNA (ceRNA) that effectively sequesters miR‐30c‐5p, thereby alleviating its post‐transcriptional repression of P4HA2 and resulting in P4HA2 upregulation. As a collagen‐modifying enzyme, the overexpressed P4HA2 catalyses collagen hydroxylation, leading to extracellular matrix (ECM) crosslinking and tissue stiffening. It enhances tumour cell mechanical invasiveness and drives epithelial‐to‐mesenchymal transition (EMT), facilitating lymphatic intravasation. As miR‐30c‐5p typically acts as a tumour‐suppressive miRNA, its downregulation may derepress metastasis‐associated genes, indirectly promoting lymph node metastasis.

In summary, our study demonstrated that LINC00240 regulates the proliferation, migration, and invasion of ovarian cancer cells through the miR‐30c‐5p/P4HA2 axis, providing new insights into the biology of ovarian cancer. This finding not only highlights the significance of lincRNAs and miRNAs in tumor progression but also suggests potential new biomarkers for the early diagnosis and prognosis assessment of ovarian cancer. Furthermore, intervention strategies targeting this regulatory axis may serve as new therapeutic targets, offering more personalised and effective treatment options for ovarian cancer patients.

## 5 Conclusions

Our findings suggest that LINC00240 is significantly elevated in ovarian cancer tissues. Knockdown of LINC00240 suppresses the aggressive behaviours of OVCAR3 and SKOV3 cells. In addition, LINC00240 directly targets miR‐30c‐5p, and blocking miR‐30c‐5p could reverse the hindered proliferation and migration ability induced by LINC00240 knockdown. Meanwhile, miR‐30c‐5p targets P4HA2. Overall, the LINC00240/miR‐30c‐5p/P4HA2 signalling pathway appears to be instrumental in managing ovarian cancer progression, and it may act as an encouraging treatment target for interventions in ovarian cancer.

## Author Contributions

**HaiBo Zhang:** writing – review and editing, conceptualization, funding acquisition, supervision. **Yunjie Tian:** conceptualization, methodology, writing – original draft, software, data curation. **Jianlei Wu:** methodology, funding acquisition, data curation, visualization.

## Funding

This work was supported by the Medical Science Research Project of Hebei (No. 20220164 and 20240233) and Youth Science Foundation Project of Shandong First Medical University (No. 202201‐116).

## Conflicts of Interest

The authors declare no conflicts of interest.

## Supporting information


**Table S1:** Basic pathological information of the patient.

## Data Availability

The data that support the findings of this study are available from the corresponding author upon reasonable request.
